# Readability and content analysis of lifestyle education resources for weight management in Australian general practice

**DOI:** 10.1186/s40608-016-0097-1

**Published:** 2016-03-09

**Authors:** Nouhad El-Haddad, Catherine Spooner, Nighat Faruqi, Elizabeth Denney-Wilson, Mark Harris

**Affiliations:** Centre for Primary Health Care and Equity, UNSW Australia, New South Wales, Australia; Centre for Obesity Management and Prevention Excellence in Primary Health Care, UNSW Australia, New South Wales, Australia; Faculty of Health, University of Technology Sydney, New South Wales, Australia

**Keywords:** Educational resources, Weight management, Lifestyle, Nutrition, Physical activity, Health literacy, Readability, Content analysis, General practice

## Abstract

**Background:**

Weight management education is one of the key strategies to assist patients to manage their weight. Educational resources provide an important adjunct in the chain of communication between practitioners and patients. However, one in five Australian adults has low health literacy. The purpose of this study was to assess the readability and analyse the content of weight management resources.

**Methods:**

This study is based on the analysis of 23 resources found in the waiting rooms of ten Sydney-based general practices and downloaded from two clinical software packages used at these practices. The reading grade level of these resources was calculated using the Flesch Reading Ease, Flesch-Kincaid Grade Level, Fry Readability Graph, and the Simplified Measure of Gobbledygook. Resources’ content was analysed for the presence of dietary, physical activity, and behaviour change elements, as recommended by *the Clinical practice guidelines for the management of overweight and obesity in adults, adolescents, and children in Australia.*

**Results:**

The resources’ average reading grade level was for a 10^th^ grader (9.5 ± 1.8). These findings highlight that the average reading grade level was two grades higher than the recommended reading grade level for health education resources of 8th grade level or below. Seventy percent of resources contained dietary and behaviour change elements. Physical activity was included in half of the resources. Two messages were identified to be inconsistent with the guidelines and three messages had no scientific basis.

**Conclusion:**

A body of evidence now exists that supports the need to develop evidence-based education resources for weight management that place low demand on literacy, without compromising content accuracy. The findings from this study suggest that there is significant room for improvement in the educational resources provided in general practices.

## Background

Obesity is a major global health concern. In Australia, obesity is common among general practice patients [1]. Approximately 70 % of Australian men and 56 % of Australian women were classified as overweight or obese in 2012 [2].

The Australian National Health and Medical Research Council (NHMRC) *Clinical practice guidelines for the management of overweight and obesity in adults, adolescents, and children in Australia* (the NHMRC Guidelines) provide recommendations for obesity management in general practice [3]. According to these guidelines, patient education should encourage healthy nutrition that create a required energy deficit, increased physical activity and strategies for behaviour change [4]. These lifestyle interventions are generally the first approach used by general practitioners (GPs) in assisting patients to manage their weight [3, 5]. GPs are encouraged to educate their patients on weight loss and lifestyle change [6–13]. This can provide support for patient’s behavioural change when combined with other interventions [6–8, 10–14].

Patient educational resources for weight management can provide a cost-effective way to reinforce verbal information provided by health professionals and promote behaviour change in overweight patients [3]. However, in Australia, one in five adults do not have adequate health literacy skills to understand health information [2, 15] and use it to make suitable changes to their weight [15]. Patients with better knowledge of their condition and its management have better outcomes [4]. Educational resources could increase patient’s knowledge and understanding of weight management if they met the reading ability of the general population [15]. One of the elements for comprehending text is readability, which refers to the understandability of written text [16]. Readability can be calculated using a formula that determines the reading ability needed to understand a piece of text [16]. South Australia (SA) Health recommend that the level of readability of health information should be at a reading level of an eighth grader [17], which is the level of a 13 to 16 year old with 8 years of Australian education [18].

To our knowledge, no previous research has assessed the readability and analysed the content of lifestyle educational resources for weight management available for patients of the Australian primary health care system. A Canadian study assessed the readability of physical activity educational resources and found that the readability of the resources were above the average reading ability of adults (grade 10 or above) [15]. Studies on other health topics reported similar results. These include studies on paediatric care [19, 20], mental health [21], medication side effects and complications [22], hay fever [23], sleep disorders [24], stroke rehabilitation [25, 26], anticoagulant therapy [27], cholesterol [28], oral health and skin care for pressure ulcers [29].

The primary aim of this study was to assess the reading grade level of lifestyle educational resources for weight management available in Australian general practices. The secondary aim of this study was to analyse the content of the education resources in relation to the NHMRC Guidelines*.*

## Methods

Lifestyle educational resources for weight management that included nutrition and/or physical activity information, were collected from ten general practices in South Western Sydney recruited as part of a cluster randomised control trial [30]. The practices served a diverse population that included people from non-English speaking and socioeconomically disadvantaged backgrounds, with obesity and low health literacy. The aim of trial was to evaluate a multi-level intervention for obese patients with low health literacy attending primary health care. The trial’s protocol has been previously published [30]. Educational resources, which satisfied the eligibility criteria were collected from the practices’ waiting rooms. Additionally appropriate resources were identified from the two clinical management software used in these practices. In Australia, these software’s are commonly used by general practices to manage patients’ clinical information, drug prescription and are a source for patient educational resources.

Educational resources included in this study were published in English, targeted patients aged 18 years or older and aimed at providing lifestyle education for weight management, including nutrition and/or physical activity information. Educational resources were excluded if they provided nutrition and/or physical information not specifically related to weight management, including pregnancy, breastfeeding, children, adolescents, allergies, intolerances, cancer, gastrointestinal disorders and any chronic condition including cardiovascular disease and diabetes.

The first author (NEH) assessed every educational resource recorded in the software packages and located in the waiting rooms for eligibility. The list of resources and their classification according to the inclusion criteria were verified with the co-authors (CS, NF and MH) and discrepancies were discussed between the researchers.

### Readability analysis

**Fig. 1:**
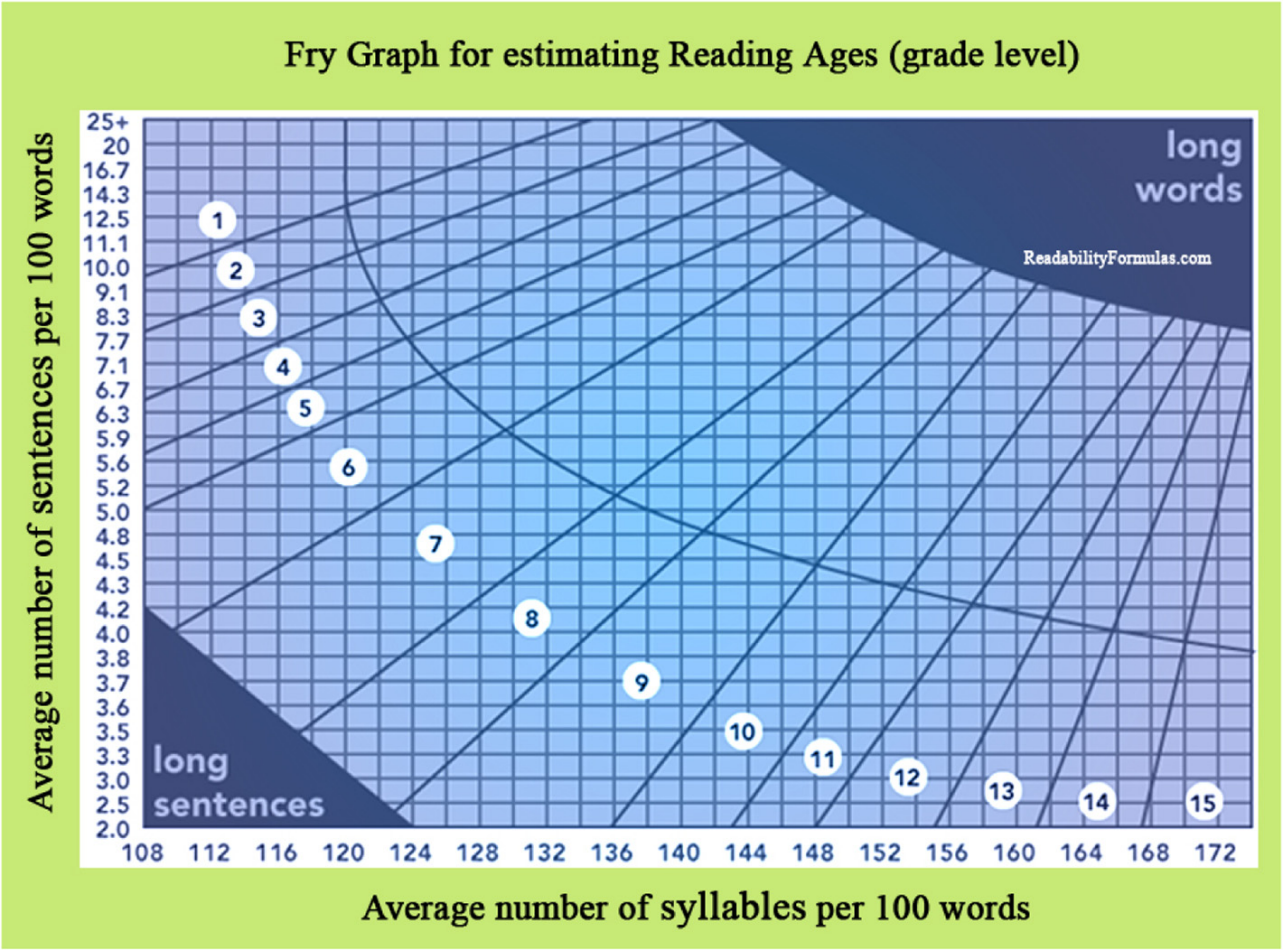
Fry Readability Graph for estimating the reading grade level [33]

The readability of included resources was determined by the use of Flesch Reading Ease score [31], Flesch-Kincaid Grade Level (FKGL) [32], Fry Readability Graph [33] and the Simplified Measure of Gobbledygook (SMOG) Grade [34]. The Flesch Reading Ease score and the FKGL were used because of their widespread use and validation for calculating readability [16, 31, 32]. Fry Readability Graph and SMOG were selected because both are commonly used and are suitable to assess the readability of patient educational material and health information [35]. These readability tools have been previously validated [16]. The Flesch Reading Ease score is based on the average number of words in the sentence and the average number of syllables per 100 words [31]. The Flesch Reading Ease formula produces a reading score between 0 and 100. A higher score means that the text is considered easier to understand; whereas a lower score indicates a more difficult text to read [31]. A reading ease score between 60 and 70 is considered acceptable [31]. The Flesch Reading Ease score was converted to a reading grade level (Table 1) and compared with reading grade levels calculated with the other readability tools [32]. The FKGL is based on the average number of words per sentence and the average number of syllables per word [32]. SMOG Grade is based on the square root of the number of words with three or more syllables per 30 sentences [34]. SMOG Grade is considered the gold standard for evaluating health information [16]. The scores from FKGL and SMOG Grade corresponds to the number of formal school years necessary to understand the text [32]. Fry Readability Graph is based on the average number of sentences per 100 words and the number of syllables per 100 words [33]. Average scores were plotted onto the Fry Graph (Fig. 1). The intersection of the average number of sentences and the average number of syllables lines indicates the average reading grade level of the text.

**Table 1:** Interpretation of the reading ease formula [31]

Reading grade level	Verbal description	Reading ease
5	Very easy	90–100
6	Easy	80–89
7	Fairly easy	70–79
8–9	Standard	60–69
10–12	Fairly difficult	50–59
13–16	Difficult	30–49
Above 16	Very difficult	0–29

Flesch Reading Ease and FKGL predict the reading grade level based on 75 % comprehension, Fry Readability Graph predicts the reading grade level based on 90 % comprehension, while SMOG Grade predicts the reading grade level based on 100 % comprehension and complete understanding of health information. The reading grade levels yielded from the four readability tools were compared. The reading grade levels are based on US grade levels, which are equivalent to Australian grade levels [18].

The title and content of eligible resources were pasted into an automated online program as plain text (i.e. without pictures, bullets, abbreviations, and text boxes) that calculated performed the various formulas [36]. To check for accuracy, the word and sentence count obtained by the online program were manually confirmed.

Readability test data were entered and analysed descriptively for mean and standard deviation (SD) values using the SPSS Statistical Package, version 22.0 (SPSS INC., Chicago, IL, USA).

### Content analysis

Each identified educational resource was assessed for the presence or absence of three overarching content elements: diet; physical activity; and behaviour change.

The three dietary content elements were: advice to eat more of the healthy food groups; limit intake of high-energy foods and drinks; and reduce total energy intake. These elements were chosen because the NHMRC Guidelines and the Australian Dietary Guidelines [3, 37] recommend that dietary advice should focus on creating an energy deficit and include a wide variety of nutritious foods.

The three physical activity elements were: advice to participate in approximately 300 min of moderate-intensity activity; 150 min of vigorous activity; or an equivalent combination of moderate-intensity and vigorous activity each week combined, in conjunction with reduced dietary intake. These elements were chosen because *the* NHMRC Guidelines recommend these levels of physical activity for adults with overweight or obesity [3].

The five behavioural change content elements were: goal setting; self-monitoring of behaviour and progress; stimulus control; cognitive restructuring; and problem solving. These elements were chosen because the NHMRC Guidelines include the following examples of core strategies to support behavioural change for weight management [3].

Messages that were ambiguous, confusing or conflicted with the NHMRC Guidelines were noted for further investigation.

## Results

Twenty-three educational resources were included in this study (Table 2). The majority of the educational resources were from commercial sources (*n* = 15), seven were from not-for-profit organisations and one was from a government source. Of the 23 resources, most (*n* = 17) were published between 2004 and 2014. Six resources did not include the date of publication. One of the resources was discontinued from publishing since 2010. Of the 23 resources, six were directly related to body weight and weight management, eight were focused on nutrition, six on physical activity, and three included information on a general healthy lifestyle.

**Table 2:** Reading grade level estimates and descriptive data of included resources

No.	Title	Flesch reading ease	FKGL^a^	Fry Readability	SMOG^b^ Grade	Average	Standard deviation	Type of publisher	Year published
1	Body weight and cancer risk	7.0	8.6	9.0	10.6	8.8	1.5	Not-for-profit	2014
2	Physical Activity	10.0	11.2	14.0	12.9	12.0	1.8	Not-for-profit	2014
3	Want to get more life out of life? You can do something positive^d^	8.0	9.3	12.0	10.1	9.9	1.7	Government	2010
4	Eat for health	7.0	6.6	7.0	9.6	7.6	1.4	Not-for-profit	NA^c^
5	Move your body	8.5	7.8	9.0	10.9	9.1	1.3	Not-for-profit	NA
6	Stay in shape	7.0	7.0	7.0	10.1	7.8	1.6	Not-for-profit	NA
7	Why be active?	11.0	11.0	11.0	13.3	11.6	1.2	Not-for-profit	NA
8	Very low calorie diets: What you need to know^e^	8.0	9.2	15.0	12.4	11.2	3.2	Commercial	NA
9	Lifestyle choices for better health	11.0	10.5	10.0	13.2	11.2	1.4	Not-for-profit	2012
10	Medically supervised weight-loss program^f^	7.71	7.0	9.1	9.9	8.4	1.3	Commercial	NA
11	Healthy eating	12.0	12.8	9.0	14.4	12.1	2.3	Commercial	2005
12	Healthy takeaways	7.0	7.8	8.0	11.1	8.5	1.8	Commercial	2005
13	High protein diets	9.0	8.7	11.0	11.2	10.0	1.3	Commercial	2004
14	Reading food labels	8.5	9.7	4.0	13.2	8.9	3.8	Commercial	2005
15	The facts on fat	8.0	9.3	9.0	12.6	9.7	2.0	Commercial	2005
16	Weight control	7.0	8.2	7.0	11.7	8.5	2.2	Commercial	2005
17	Achieving an adequate diet	8.0	9.1	12.0	11.5	10.2	1.9	Commercial	2009
18	Achieving and maintaining a healthy weight	7.0	6.7	7.0	9.6	7.6	1.4	Commercial	2008
19	Dietary guidelines for healthy eating	8.0	8.5	10.0	11.4	9.5	1.5	Commercial	2009
20	Exercise: easing into it	8.0	8.6	9.0	11.6	9.3	1.6	Commercial	2006
21	Heart disease reduce the risk	7.0	5.9	8.0	9.0	7.5	1.3	Commercial	2009
22	Obesity Q and A	9.0	7.2	8.0	10.1	8.6	1.3	Commercial	2010
23	Physical activity and exercise: getting started	12.0	9.6	14.0	12.0	11.9	1.8	Commercial	2009

^a^ *FKGL* flesch-kincaid grade level

^b^ *SMOG* simplified measure of gobbledygook

^c^ *NA* not available

^d^This resource was available in English and in Arabic. It has been discontinued from publication

^e^This resource is an advertisement for a meal replacement product as part of a very low calorie diet

^f^This is a series of educational resources as part of Professor Trim’s medically supervised weight-loss program

### Readability analysis

Table 2 shows the reading grade level of the resources. Four resources, entitled “Achieving and maintaining a healthy weight”, “Eat for health”, “Heart disease reduce the risk” and “Stay in shape”, achieved the lowest reading grade level. Their average reading grade was noted at 8^th^ grade. Four resources, entitled “Healthy eating”, “Physical activity”, “Physical activity and exercise: getting started” and “Why be active”, achieved the highest average reading grade level of a 12^th^ grader. Two of these resources, “Healthy eating” and “Physical activity”, contained the most comprehensive information out of the 23 resources. One educational resource, “Achieving and maintaining a healthy weight”, placed one of the lowest literacy demands on readers (7.6 ± 1.4) and covered all of the weight management messages. Resources produced by not-for-profit organisations had a readability range from 8^th^ to 12^th^ grade (9.7 ± 1.4); the one resource produced by the government had a readability score of 10^th^ grade (9.9 ± 1.7); and those produced commercially had a readability range from 8^th^ to 12^th^ grade (9.6 ± 2.0).

The average reading grade level calculated by SMOG Grade (12 ± 1.4) was higher compared with Flesch Reading Ease (8.6 ± 1.4), FKGL (8.7 ± 1.4) and Fry Readability (9.4 ± 1.4).

### Content analysis

Table 3 shows the coverage of weight management messages for each resource. There was a broad range of messages covered. The most prevalent messages found in these resources were related to the consumption of more of the healthy food groups, reduction of total energy intake and support for behavioural change, more specifically goal setting and cognitive restructuring.

**Table 3:** Content analysis of included resources

No.	Diet									Physical activity			Behaviour change				
	Advice to eat more of the healthy food groups				Advice to limit intake of high-energy foods and drinks				Advice to reduce total energy intake	300 min of moderate-intensity activity/week	150 min of vigorous activity/week	Combination of moderate-intensity and vigorous activity each week in conjunction with reduced dietary intake	Goal setting	Self-monitoring of behaviour and progress	Stimulus control	Cognitive restructuring	Problem solving
	Vegetables and fruit	Wholegrain/high fibre cereals	Lean meat/alternatives	Reduced fat dairy products	Foods high in saturated fat^b^	Foods and drinks with added salt	Foods and drinks with added sugar^a^	Alcohol									
1	Y	Y	Y	-	-	Y	Y	Y	Y	-	-	Y	Y	Y	Y	Y	Y
2	Y	Y	Y	-	-	Y	Y	Y	Y	Y	Y	Y	Y	-	Y	Y	Y
3	Y	-	-	-	-	-	-	Y	-	Y	-	Y	Y	-	-	Y	-
4	Y	Y	Y	Y	Y	Y	Y	Y	Y	-	-	-	Y	-	-	-	-
5	-	-	-	-	-	-	-	Y	-	Y	Y	Y	Y	-	Y	Y	Y
6	Y	Y	Y	Y	Y	Y	Y	-	Y	-	-	Y	Y	Y	Y	Y	Y
7	-	-	-	-	-	-	-	-	-	Y	-	-	Y	-	Y	Y	Y
8	-	-	-	-	-	-	-	-	Y	-	-	-	Y	Y	-	-	-
9	Y	Y	Y	Y	Y	Y	Y	Y	-	Y	-	Y	Y	Y	Y	Y	Y
10	Y	Y	Y	Y	Y	Y	Y	-	Y	Y	Y	-	Y	-	Y	Y	Y
11	Y	Y	Y	Y	Y	Y	Y	Y	Y	Y	Y	Y	Y	-	-	Y	Y
12	Y	Y	Y	-	Y	-	-	Y	Y	-	-	-	Y	-	Y	Y	-
13	Y	Y	Y	-	-	-	Y	-	-	-	-	-	Y	-	Y	Y	-
14	-	Y	-	Y	Y	Y	Y	-	Y	-	-	-	Y	-	-	Y	Y
15	-	-	Y	Y	Y	-	-	-	Y	-	-	-	Y	-	-	Y	-
16	Y	Y	Y	Y	Y	Y	Y	-	Y	Y	Y	Y	Y	-	Y	Y	Y
17	Y	Y	Y	Y	Y	Y	Y	Y	Y	-	-	-	-	-	-	-	-
18	Y	Y	Y	Y	Y	Y	Y	Y	Y	-	-	Y	Y	Y	Y	Y	Y
19	Y	Y	Y	Y	Y	Y	Y	Y	Y	Y	-	Y	Y	-	Y	Y	Y
20	-	-	-	-	-	-	-	-	-	Y	Y	-	Y	-	Y	Y	Y
21	Y	Y	Y	Y	Y	Y	Y	Y	Y	-	-	Y	Y	-	Y	Y	Y
22	Y	Y	Y	Y	Y	Y	Y	Y	Y	Y	Y	Y	Y	Y	Y	Y	Y
23	-	-	-	-	-	-	-	-	-	Y	Y	-	Y	-	Y	Y	Y

*Y* yes

^a^Foods and drinks with high-added sugar include confectionary, sugar-sweetened soft drinks and cordials, sugar-sweetened and no added sugar fruit drinks, vitamin waters, energy and sports drinks

^b^Food high in saturated fat include biscuits, cakes, pastries, pies, processed meats, commercial burgers, pizzas, fried foods, potato chips, crisps and other savoury snacks, butter, cream, cooking margarine, coconut and palm oil

Dietary content elements —namely advice to eat more vegetables, fruit, wholegrain or high fibre cereals, lean meat or meat alternatives and reduce total energy intake— were covered in 16 out of the 23 resources. Advice to limit intake of high-energy foods and drinks were covered in 14 resources.

Physical activity content elements —namely advice to participate in approximately 300 min of moderate-intensity activity and an equivalent combination of moderate-intensity and vigorous activity each week in conjunction with reduced dietary intake— was covered in 12 of the 23 resources. Advice to participate in 150 min of vigorous activity was covered in eight resources.

Behavioural change content elements —namely goal setting and cognitive restructuring— were covered in most resources (96 and 87 % respectively). Stimulus control and problem solving were covered in 16 resources. A quarter (26 %) of the resources contained information on self-monitoring of behaviour and progress.

Two resources, “Achieving and maintaining a healthy weight” and “Obesity Q and A”, provided the most comprehensive information, covering all 17 weight management messages. One resource, “Very low calorie diets: What you need to know”, provided the least amount of information, covering only three messages.

Two messages that were inconsistent with the NHMRC Guidelines were identified. These were included in two resources, “Body weight and cancer risk” and “Medically supervised weight-loss program”. They asserted that diets were ineffective and/or counter-productive. Additionally, one resource*,* “Medically supervised weight-loss program”, contained multiple pieces of information that had no scientific basis. For example, it recommended using spicy foods and caffeine to reduce hunger and recommended slow weight loss as this approach prevented the negative ‘bounce back’ phenomenon said to be associated with sudden weight loss.

## Discussion

Weight management education is one of the key strategies to assist patients to manage their weight [6–13]. Educational resources provide an important adjunct in the chain of communication between practitioners and patients. This study found that the average reading grade level was two grades higher than the recommended reading grade level for health education resources of 8^th^ grade level or below [17]. The average reading grade level calculated by SMOG Grade was four grades higher than the recommended reading grade level. The average reading grade level calculated by Flesch Reading Ease and FKGL was one grade higher and the average reading grade level from Fry Readability Graph was two grades higher than the recommended reading grade level.

Our findings suggest that these resources would place high demands (reading grade level of a 12^th^ grader) on patients to understand all the information. These resources are likely to be difficult to comprehend for patients with low health literacy skills. The content analysis indicated that there was consistent information between the majority of the resources and the NHMRC Guidelines recommendation. The most prevalent messages found in the resources related to the consumption of more foods from the healthy food groups, reduction of total energy intake and support for behaviour change, specifically goal setting and cognitive restructuring. Only half of the resources covered some physical activity content elements. The NHMRC Guidelines strongly recommend a multicomponent intervention that incorporates reduced energy intake, increased physical activity and measures to support behaviour change [3, 5]. Resources designed for weight management should also include information on physical activity.

Some messages were found to be inconsistent with the NHMRC Guidelines recommendations. Despite evidence that diets are effective for weight management [3], two resources asserted that diets were ineffective or counter-productive. Dietary interventions should be incorporated into a weight loss intervention and are designed to create an overall caloric deficit, suited to the individuals’ needs and preferences [4]. The degree of adherence to the diet is the principal determinant of weight loss [3]. Another resource recommended slow weight loss as opposed to rapid weight loss to prevent the ‘bounce back’ phenomenon. However, a recent randomised control trial found that the rate of weight loss did not affect the proportion of weight regained within 144 weeks [38]. Also, rapid weight loss has a motivating effect, as seen in patients on a very low energy diet, although it is difficult to maintain in the long-term [39].

A number of claims were unsupported by published literature. A resource aimed at providing tips for reducing hunger suggested that consuming spicy food and caffeine before a main meal could act as a hunger-reducing appetiser for weight loss. Caffeine has been found to aid weight loss because of its thermogenic effect [40, 41]. A meta-analysis assessed the impact of green tea catechins (GTCs) with or without caffeine on body weight and found that GTCs with caffeine significantly decreased body weight when compared with a caffeine-free control [42]. However, the clinical significance of weight loss was modest [42]. Resources that recommend the consumption of caffeine to reduce hunger and assist with weight loss run the risk of encouraging unsafe caffeine use. For a healthy adult, caffeine intake exceeding 400 mg per day is associated with adverse effects, such as general toxicity, insomnia, cardiovascular effects, effects on bone status and calcium balance, negative fluid balance caused by diuresis, changes in adult behaviour, increased incidence of cancer and effects of male fertility [43]. In relation to the spicy food claim, capsaicins, the pungent or hot compound in chilli peppers [44], are theorised to aid in weight loss because of their potential ability to increase energy expenditure and fat oxidation and decrease appetite. However, short-duration clinical trials have not demonstrated that capsaicins in spicy food are effective for weight loss among overweight or obese individuals [44–47]. Any effect on resting metabolic rate was approximately 50 cal per day and not statistically significantly different to those in the placebo group [44, 45]. The long-term efficacy of spicy food to aid in weight loss has, therefore, not yet been tested.

The present study has several limitations. It is not an exhaustive study of resources available for weight management for patients attending general practice. The study only included educational resources published in English and those collected from medical software packages and the waiting room of the selected practices. The study did not include resources that GPs might download from the Internet or other sources. Second, the appraisal of a small sample of local general practices makes it difficult to generalise our findings as the results may underrepresent Australian practices. There may be resources used in other Australian practices with different readability and content. The authors also recognised that new resources continue to be developed. Third, other aspects of general readability were not assessed. These included the use of textual characteristics, such as font size, colour, bold and italics text to emphasise information, bulleted text, as well as instructional pictures, simplified sentences, sentence length, active and passive verbs and white space [48, 49]. Finally, the current study did not seek the opinion of the patients. This could have provided useful information on the acceptability, suitability, usefulness and understanding of the educational resources. Hence, further studies that analyse the readability and content of a larger sample of weight management education resources and that evaluate the impact of these resources are recommended.

## Conclusion

A body of evidence now exists that supports the need to develop evidence-based education resources for weight management that place low demand on literacy, without compromising content accuracy. This study suggests that educational resources commonly available in Australian general practice fall short of this. This has implications for general practice accreditation, which requires general practices to provide up-to-date resources of high quality and reliability for patients [50].

## Abbreviations

FKGL: flesch-kincaid grade level
GPs: general practitioners
GTC: green tea catechins
NHMRC: National Health and Medical Research Council
SA: South Australia
SD: standard deviation
SMOG: simplified measure of gobbledygook
SPSS: statistical package for the social sciences
